# Supplementary material for: E4BP4 in macrophages induces an anti-inflammatory phenotype that ameliorates the severity of colitis
Source: Commun Biol. 2024 May 7;7:527. doi: 10.1038/s42003-024-06099-4 (PMC11076557; doi:10.1038/s42003-024-06099-4)
Supplement: Supplementary file 2 — Supplementary Information [file 42003_2024_6099_MOESM2_ESM.pdf]

Supplementary Table 1. # M2marker gene lists

Arg1	C630043F03Rik	6430531B16Rik	Amd2	Timm8a1
Cd207	Slamf6	Tmem158	Rcn3	Snhg5
H2-Eb1	Lrp4	P2ry12	Fgd1	Ccl24
Sertad4	Smap2	Fhad1	Azin2	
Gnat3	Gpr155	Efnb1	Ebf3	
Mrc1	Lifr	Ube2cbp	Zfp808	
Olfr110	Bcar3	Clcn5	Gm20319	
H2-M5	Plekha3	Zbed5	Hacd1	
Postn	Fjx1	Dmpk	Ski	
Slc7a14	Gm5150	Hfe	Mir8098	
Mir6990	Wdfy2	Usp2	Slc5a6	
Egr2	Rgcc	Brwd1	Pald1	
Hbegf	Gpr183	Skida1	D5Ertd605e	
P2ry1	Awat2	Nkx6-2	Pgm2l1	
Itgb3	Slc46a3	Sh2b2	Timm9	
Abcd2	Rasl11b	Cntf	Apoe	
Pxdc1	Tgfbi	Tanc2	Zc3h8	
H2-Ab1	Axin2	Arl15	Tfap4	
Myc	Il10	Tle1	Arap2	
Mgl2	Lpxn	Dancr	Fyn	
Clec7a	Arhgap12	Tgfbr1	Gclc	
Eng	Znf41-ps	Angptl2	Tbc1d16	
Klhl33	Map2	Pkp2	Slc16a1	
Tlr8	Slc29a2	Inpp5a	Igfbp7	
Fcrls	Hpdl	Clec10a	Gnl3	
Lipn	D4Ertd617e	Snhg4	Ldlrad3	
Pparg	Zfp932	Slc18a1	Pus7	
Flt1	Cnr2	Trmt61a	Dhrs9	
Pim3	Clec4a3	Grhl1	Smpd3	
Cdcp1	Mir17hg	Amd1	Pla2g4c	

Supplementary Table 2. List of qPCR primers in this study

	forward (5' to 3')	reverse (5' to 3')
E4bp4	GGAGCAGAACCACGATAACC	TTCCCCAGTCTTCTTTCAGG
E4bp4 (for E4BP4-KO RAW 264.7)	TGGATGAGAGGCTGAAGTCC	CTGGGAGAAGAAAATGCCAC
Cd79a	GCTGCTGCTATTCAGGAAACG	AGGTTCAGGCCCTCATAGAGA
Cd3e	AGGACGATGCCGAGAACATT	CTCGTCACTGTCTAGAGGGC
Csf1r	GACCTACCGTTGTACCGAGC	AAGAGTGGGCCGGATCTTTG
S100a9	AGCACAGTTGGCAACCTTTATG	AAGCTCAGCTGATTGTCCTGG
Flt3	AGCACAGTTGGCAACCTTTATG	AAGCTCAGCTGATTGTCCTGG
Itga2	ATGGTGGGGACCTCACAAAC	GCCATCGGTCACAACTACCA
Il4ra	ACGTGGTACAACCACTTCCA	GAACAGGCAAAACAACGGGA
Gapdh	CCGCATCTTCTTGTGCAGTG	ATGAAGGGGTCGTTGATGGC

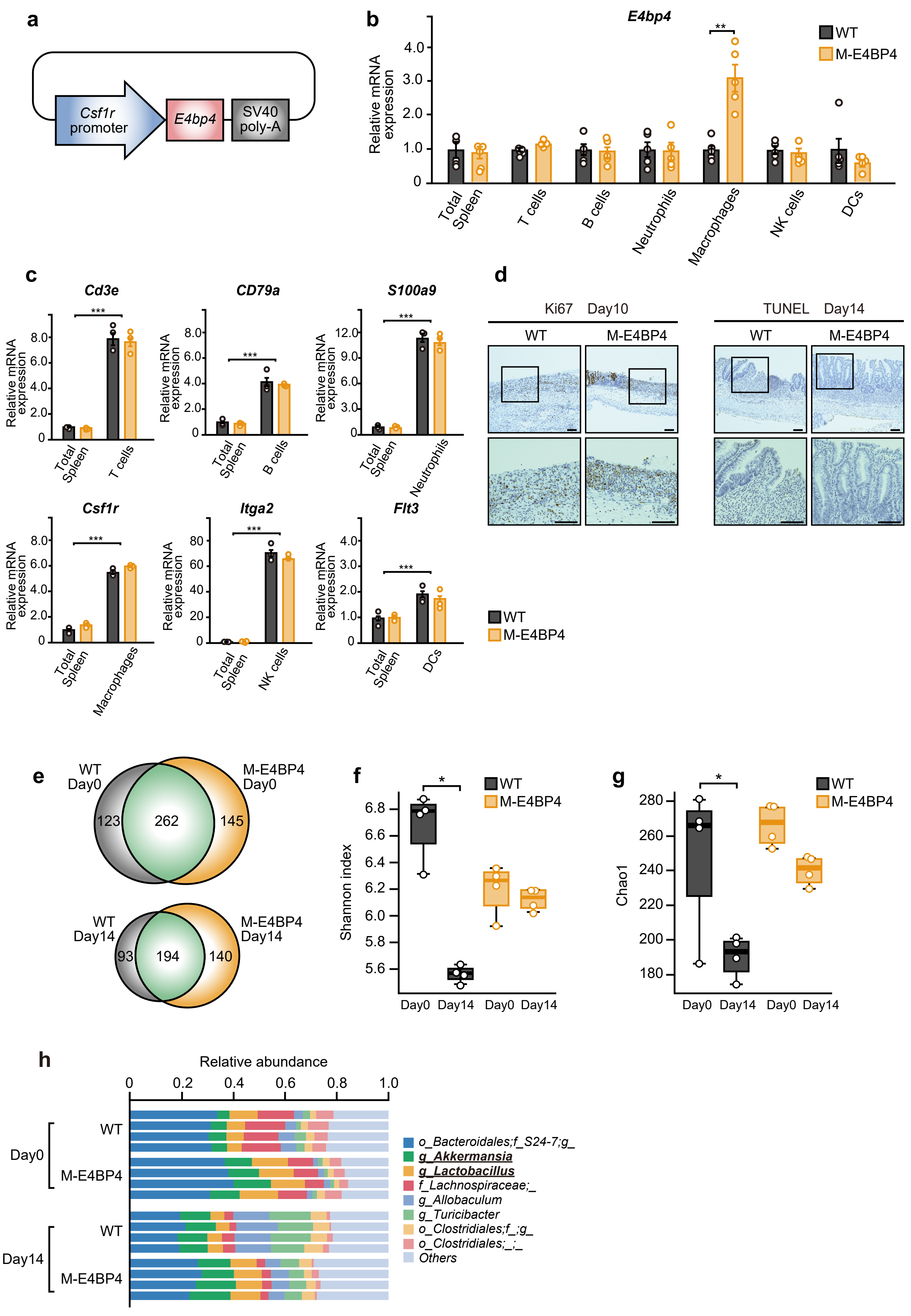
a
Csf1r promoter
E4bp4
SV40 poly-A
b
E4bp4
Relative mRNA expression
WT
M-E4BP4
**
Total Spleen
T cells
B cells
Neutrophils
Macrophages
NK cells
DCs
c
Cd3e
CD79a
S100a9
Csf1r
Itga2
Flt3
***
Relative mRNA expression
Total Spleen
T cells
B cells
Neutrophils
Macrophages
NK cells
DCs
WT
M-E4BP4
d
Ki67 Day10
TUNEL Day14
WT
M-E4BP4
e
WT Day0
M-E4BP4 Day0
123
262
145
WT Day14
M-E4BP4 Day14
93
194
140
f
Shannon index
*
WT
M-E4BP4
Day0
Day14
g
Chao1
*
WT
M-E4BP4
Day0
Day14
h
Relative abundance
Day0
Day14
WT
M-E4BP4
o_Bacteroidales;f_S24-7;g_
g_Akkermansia
g_Lactobacillus
f_Lachnospiraceae;_
g_Allobaculum
g_Turicibacter
o_Clostridiales;f_;g_
o_Clostridiales;_;_
Others

Supplementary Fig. 1 Confirmation of E4BP4 overexpression in the M-E4BP4 mice and comparison of fecal microbial composition

(a) Schema of the *E4bp4* transgene construct designed to generate M-E4BP4 mouse.

(b) *E4bp4* mRNA expression levels (n=5 in each group) in hematopoietic cells in splenic tissue. CD3, CD45R/B220, Ly6g/c, F4/80, CD49, and CD11c antibodies were used for the enrichment of T cells, B cells, neutrophils, macrophages, NK cells, and dendritic cells (DC), respectively.

(c) Confirmation of cell-specific mRNA expression (n=3 in each group). Enrichment of T cells, B cells, neutrophils, macrophages, NK cells, and DC was confirmed by expression of *Cd3e*, *Cd79a*, *S100a9*, *Csf1r*, *Itga2*, and *Flt3* genes, respectively. Significant differences were compared by summing WT and TG data against the total for *Cd3e*, *Cd79a*, *S100a9*, *Csf1r*, *Itga2*, and *Flt3*.

(d) Representative images of longitudinal colon sections with Ki67 staining on day 10 and TUNEL staining on day 14. Scale bars: 100 μm.

(e) Venn diagram of shared and independent bacterial operational taxonomic units (OTUs) on day 0 or day 14 in WT vs M-E4BP4 mice (n=4).

(f) Comparison of Shannon index on day 0 vs day 14 in WT and day 0 vs day14 in M-E4BP4 mice (n=4, respectively).

(g) Comparison of Chao1 on day 0 vs day 14 in WT and day 0 vs day 14 in M-E4BP4 mice (n=4, respectively).

(h) Relative abundances of 16S rRNA gene sequences according to their taxonomic classification.

All values are expressed as means, error bars reflect SD. Significance was determined by two-way repeated-measures ANOVA, followed by Tukey's post-test for Figure b, c, f, g (★$p < 0.05$ ★★$p < 0.01$, ★★★$p < 0.001$).

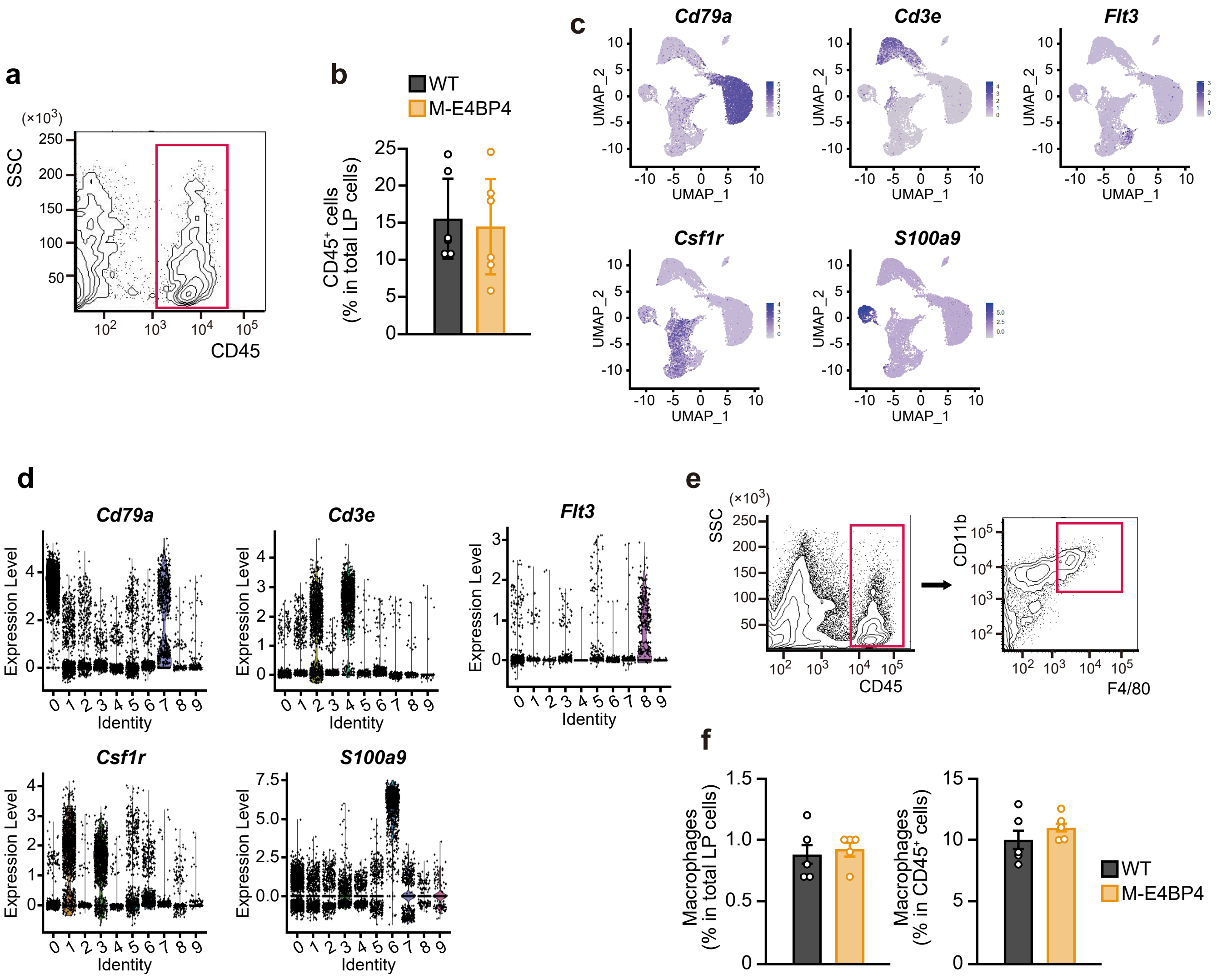
a
(×10³)
SSC
250
200
150
100
50
10²
10³
10⁴
10⁵
CD45
b
WT
M-E4BP4
CD45⁺ cells
(% in total LP cells)
25
20
15
10
5
0
c
Cd79a
Cd3e
Flt3
Csf1r
S100a9
UMAP_2
UMAP_1
d
Cd79a
Cd3e
Flt3
Csf1r
S100a9
Expression Level
Identity
e
(×10³)
SSC
CD45
CD11b
F4/80
f
Macrophages
(% in total LP cells)
Macrophages
(% in CD45⁺ cells)
WT
M-E4BP4

Supplementary Fig. 2 Single-cell analysis and bulk RNA sequencing of colon lamina propria isolated from dextran sulfate sodium (DSS)-induced colitis mice, related to Figure 2

(a) SSC and CD45 gating were used for isolating hematopoietic cells.

(b) Percentage of $CD45^{+}$ cells in total LP (lamina propria) cells in the flow cytometric analysis (n=6). Values are expressed as means, error bars reflect SD. Significance was determined by Welch's t-test.

(c) Five marker gene expression levels were overlaid on the UMAP plot. The expression levels are shown as log2(counts+1)-transformed values, and the gradient represents low (gray) to high values (purple). Each cluster was annotated using representative gene markers; *Cd79a* (B cells), *Cd3e* (T cells), *Csf1r* (macrophages), *S100a9* (neutrophils), *Flt3* (dendritic cells).

(d) Violin plots represent expression levels of five marker cytokine genes. Each cluster was annotated using representative gene markers; *Cd79a* (B cells), *Cd3e* (T cells), *Csf1r* (macrophages), *S100a9* (neutrophils), *Flt3* (dendritic cells).

(e) SSC and CD45 gating were used to select hematopoietic cells at day 14 (recovery phase). CD11b and F4/80 were used as macrophage markers.

(f) Percentage of colonic macrophages in total LP cells or CD45+ cells in the flow cytometric analysis (n=5). Values are expressed as means, error bars reflect SD. Significance was determined by Welch's t-test.

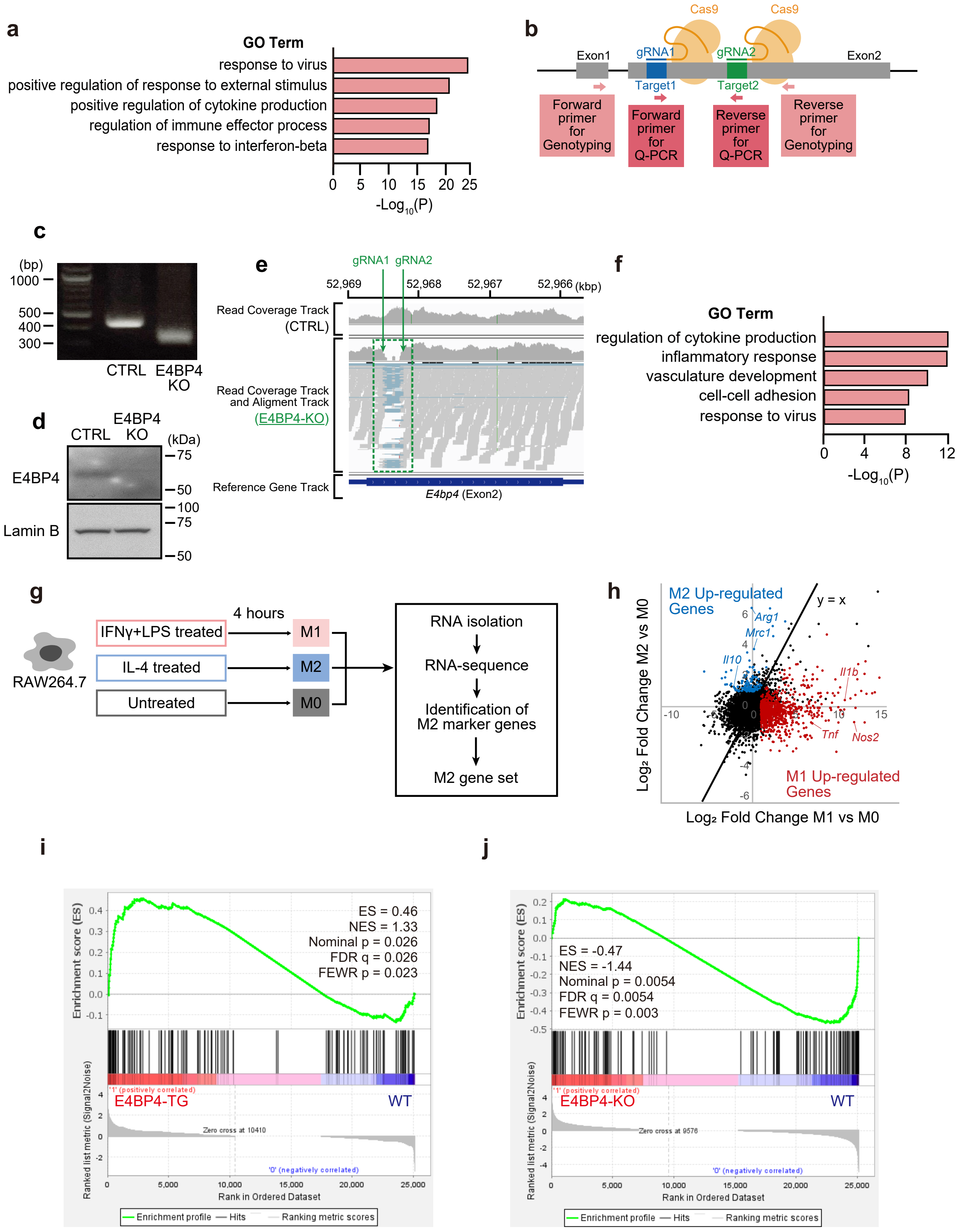
a
GO Term
response to virus
positive regulation of response to external stimulus
positive regulation of cytokine production
regulation of immune effector process
response to interferon-beta
0 5 10 15 20 25
-Log10(P)
b
Cas9
Cas9
Exon1
gRNA1
gRNA2
Exon2
Target1
Target2
Forward primer for Genotyping
Forward primer for Q-PCR
Reverse primer for Q-PCR
Reverse primer for Genotyping
c
(bp)
1000
500
400
300
CTRL
E4BP4 KO
d
E4BP4
CTRL
KO
(kDa)
75
50
100
75
50
E4BP4
Lamin B
e
gRNA1 gRNA2
52,969
52,968
52,967
52,966 (kbp)
Read Coverage Track (CTRL)
Read Coverage Track and Aligment Track (E4BP4-KO)
Reference Gene Track
E4bp4 (Exon2)
f
GO Term
regulation of cytokine production
inflammatory response
vasculature development
cell-cell adhesion
response to virus
0 4 8 12
-Log10(P)
g
RAW264.7
IFNγ+LPS treated
IL-4 treated
Untreated
4 hours
M1
M2
M0
RNA isolation
RNA-sequence
Identification of M2 marker genes
M2 gene set
h
M2 Up-regulated Genes
y = x
Arg1
Mrc1
Il10
Il1b
Tnf
Nos2
M1 Up-regulated Genes
Log2 Fold Change M2 vs M0
Log2 Fold Change M1 vs M0
i
ES = 0.46
NES = 1.33
Nominal p = 0.026
FDR q = 0.026
FEWR p = 0.023
Enrichment score (ES)
E4BP4-TG
WT
Zero cross at 10410
'1' (positively correlated)
'0' (negatively correlated)
Ranked list metric (Signal2Noise)
Rank in Ordered Dataset
Enrichment profile
Hits
Ranking metric scores
j
ES = -0.47
NES = -1.44
Nominal p = 0.0054
FDR q = 0.0054
FEWR p = 0.003
Enrichment score (ES)
E4BP4-KO
WT
Zero cross at 9576
'1' (positively correlated)
'0' (negatively correlated)
Ranked list metric (Signal2Noise)
Rank in Ordered Dataset
Enrichment profile
Hits
Ranking metric scores

Supplementary Fig. 3 Genome-wide analysis upon deletion or overexpression of E4BP4 in RAW264.7 cells, related to Figure 3

(a) Top 5 significantly enriched GO Biological Processes in differentially expressed genes described in Figure 3b.

(b) Schema of the primer design for quantitative real-time PCR and genotyping, respectively.

(c) PCR genotyping of DNA samples from RAW264.7 cells using the genotyping primer shown in Supplementary Figure 3b.

(d) E4BP4 protein expression by Western blotting.

(e) Genome alignments of exome sequence reads of Control (CTRL) and E4BP4 knockout (E4BP4-KO) RAW264.7 cells produced by the CRISPR method. The genomic regions targeted by the two gRNAs are labeled. The upperpart of each panel shows read coverage, and the lower part of E4BP4-KO shows read alignments (gray rectangles). Intra-exon deletions are highlighted by water blue lines.

(f) Top 5 significantly enriched GO Biological Processes in obviously differentially expressed genes (absolute value of Log_2 fold change > 1).

(g) Schematic procedure for the identification of M2 marker genes.

(h) Comparison of differentially expressed genes of IFNγ + LPS treated (M1) and IL-4 treated (M2) macrophages. Fold-change (FC) vs. FC plot of M1 vs. M0 on the x-axis and M2 vs. M0 on the y-axis highlighting M1 up-regulated genes in red, M2 up-regulated genes in blue, arrows indicate representative M1 or M2 genes.

(i) Gene set enrichment analysis (GSEA) enrichment plots and heat maps of differentially expressed genes belonging to the M2 gene set associated with E4BP4 expression (WT vs E4BP4-TG).

(j) GSEA enrichment plots and heat maps of differentially expressed genes belonging to the M2 gene set associated with E4BP4 expression (WT vs E4BP4-KO).

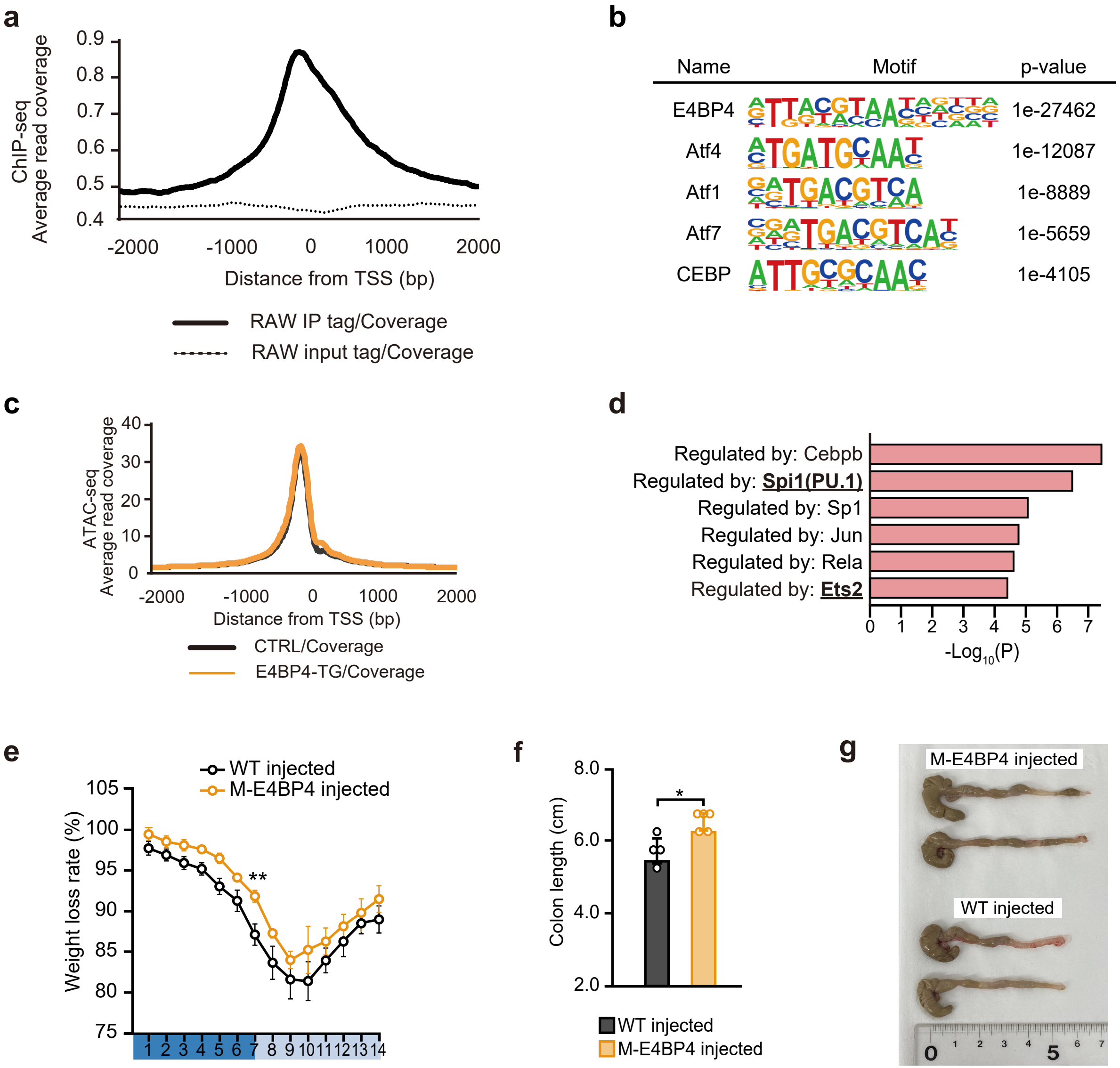
a
b
c
d
e
f
g
0.9
0.8
0.7
0.6
0.5
0.4
ChIP-seq
Average read coverage
-2000
-1000
0
1000
2000
Distance from TSS (bp)
RAW IP tag/Coverage
RAW input tag/Coverage
Name
Motif
p-value
E4BP4
1e-27462
Atf4
1e-12087
Atf1
1e-8889
Atf7
1e-5659
CEBP
1e-4105
40
30
20
10
0
ATAC-seq
Average read coverage
-2000
-1000
0
1000
2000
Distance from TSS (bp)
CTRL/Coverage
E4BP4-TG/Coverage
Regulated by: Cebpb
Regulated by: Spi1(PU.1)
Regulated by: Sp1
Regulated by: Jun
Regulated by: Rela
Regulated by: Ets2
0 1 2 3 4 5 6 7
$-Log_{10}(P)$
WT injected
M-E4BP4 injected
105
100
95
90
85
80
75
Weight loss rate (%)
**
1 2 3 4 5 6 7 8 9 10 11 12 13 14
8.0
6.0
4.0
2.0
Colon length (cm)
*
WT injected
M-E4BP4 injected
M-E4BP4 injected
WT injected
0
5

Supplementary Fig. 4 ATAC-seq and ChIP-seq of RAW264.7 cells and therapeutic effect on DSS-induced colitis mice using E4BP4-expressing macrophages

(a) Histogram of E4BP4 ChIP peak tags in WT RAW264.7 cells.

(b) Top five motifs enriched within E4BP4-ChIP peaks.

(c) Histogram of ATAC peak tags in CTRL and E4BP4-TG RAW264.7 cells.

(d) Transcription factor prediction analysis by TRRUST in upregulated genes in E4BP4-TG.

(e) Daily time courses of body weight change.

(f) Colon length on day 14 with and without 7 days' DSS treatment.

(g) Representative images of colons removed on day 14 from mice infused with CTRL or E4BP4-TG bone marrow-derived macrophage (BMDM).

All values are expressed as means, error bars reflect SD. Significance was determined by two-way repeated-measures ANOVA, followed by Tukey's post for Supplementary Figure 3d, and Welch's t-test for Supplementary Figure 4e (★$p < 0.05$, ★★$p < 0.01$).

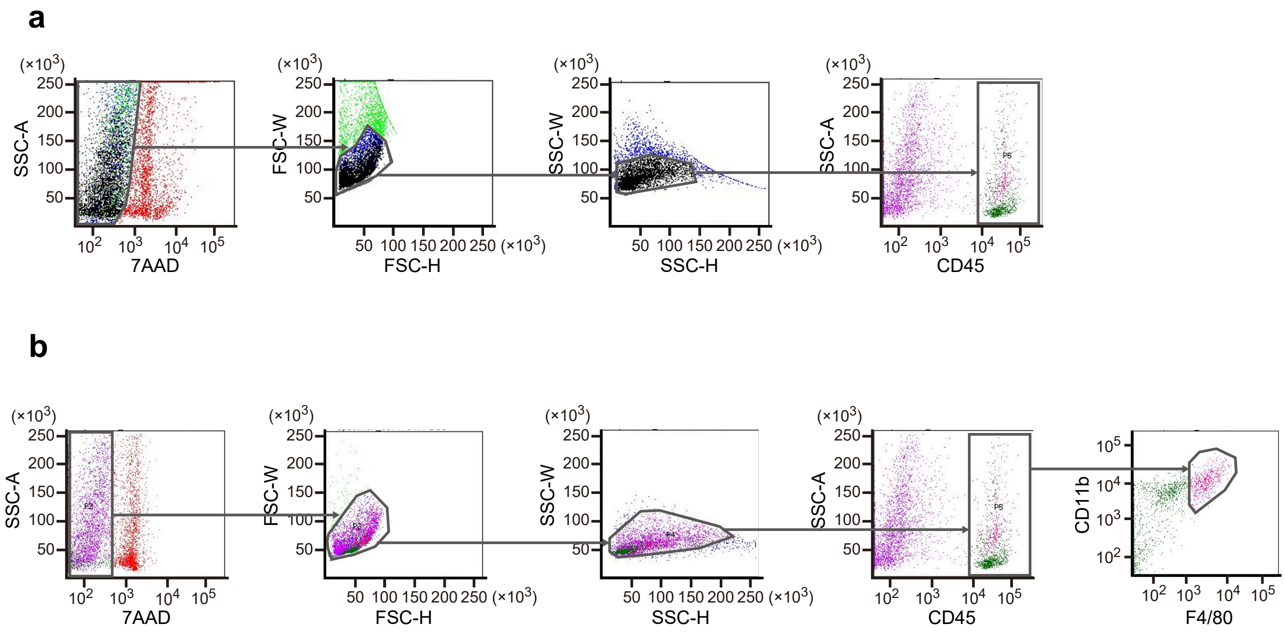
a
(×10^3)
250
200
150
100
50
SSC-A
10^2
10^3
10^4
10^5
7AAD
FSC-W
50 100 150 200 250 (×10^3)
FSC-H
SSC-W
SSC-H
CD45
b
CD11b
F4/80

Supplementary Fig. 5 Gating strategy used for flow cytometric analysis in colon lamina propria

After excluding Aminoactinomycin D (7ADD) $^{+}$ dead cells, single cells were gated based on FSC-W/FSC-H and SCC-W/SCC-H. Hematopoietic cells were gated based on CD45 expression **(a)**. Macrophages were further gated on CD11b^{+} and F4/80^{+} **(b)**.

Uncropped and unedited gel image of Supplementary Figure 3c

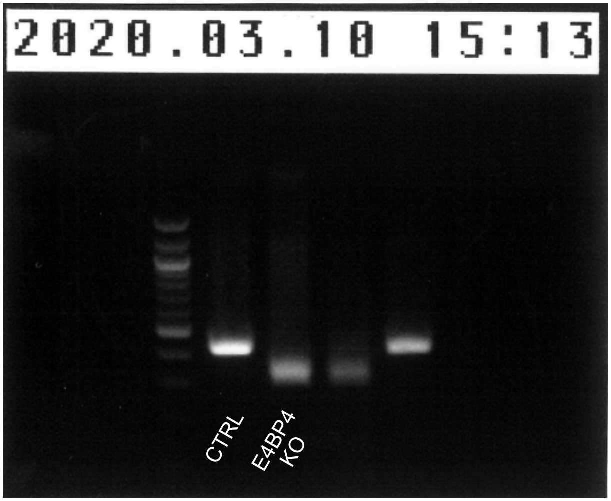

Uncropped and unedited blot image of Supplementary Figure 3d

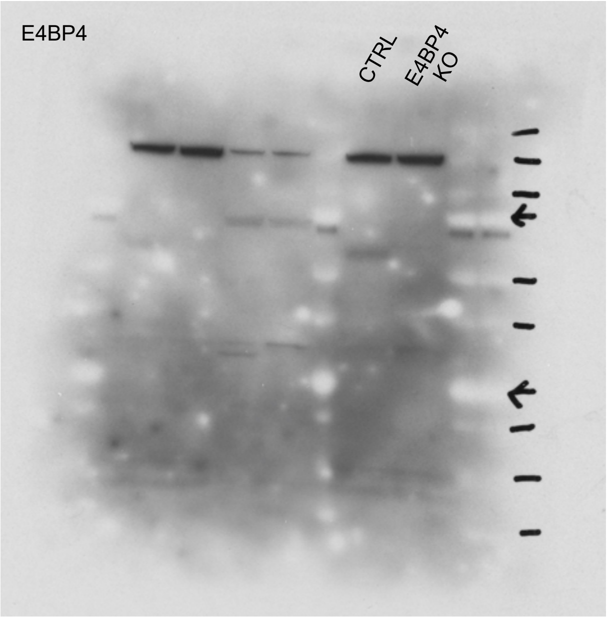

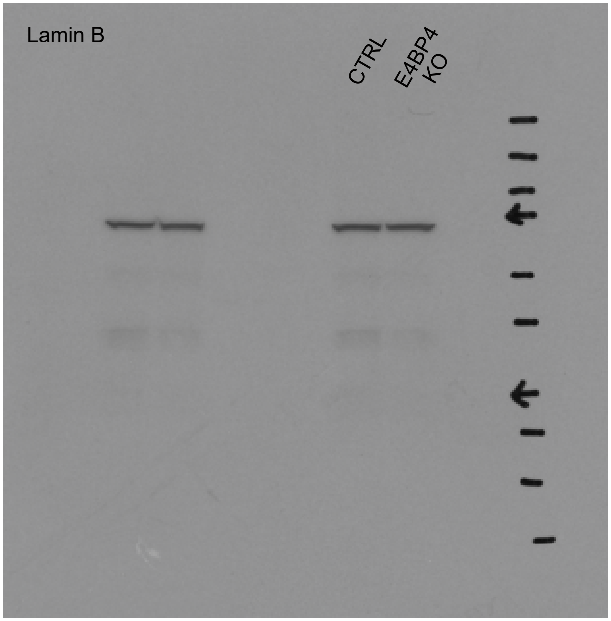

Supplementary Fig. 6 Uncropped and unedited gel image
